# From general to specific: Tailoring large language models for real‐world medical communications

**DOI:** 10.1002/ctm2.70157

**Published:** 2024-12-31

**Authors:** Xinti Sun, Wenjun Tang, Zigeng Huang, Erping Long, Peixing Wan

**Affiliations:** ^1^ State Key Laboratory of Respiratory Health and Multimorbidity Institute of Basic Medical Sciences Chinese Academy of Medical Sciences and Peking Union Medical College Beijing China; ^2^ Laboratory of Immune Cell Biology Center for Cancer Research National Cancer Institute National Institutes of Health Bethesda Maryland USA

1

The development of generative artificial intelligence (AI), such as large language models (LLMs), has garnered significant attention due to their proficiency in interpreting instructions and generating human‐resembled responses. Autoregressive LLMs, like GPT, are pre‐trained on large‐scale natural language corpora.^1^ Subsequently, they are fine‐tuned using human‐provided instructions, endowing them with generalized application capabilities across various tasks.

However, these foundation models lack training in specialized medical corpora, which limits their capability to accurately interpret the critical aspects of patient‐provider communications, leading to misinterpretation or inaccuracies in real‐world applications.^2^ Over the past 2 years, several medical LLMs have been developed or fine‐tuned using medical corpora and professional knowledge. For example, among Chinese LLMs, Sunsimiao‐7B was fine‐tuned from Qwen2‐7B with medical corpora, achieving *state‐of‐the‐art* performance in the Chinese Medical Benchmark test.^3^ Similarly, HuatuoGPT‐II, adopting a one‐stage adaptive training approach showed outstanding performance in the 2023 Chinese National Pharmacist Examination.^4^ Despite possessing extensive medical knowledge and excelling in medical examinations, these models have yet to be implemented in real‐world healthcare settings. A primary barrier is the lack of “site‐specific” knowledge, which refers to the unique protocols, workflows, and contextual information specific to each reception desk within a hospital. The indispensable site‐specific knowledge underscores the need for further refinement of foundation models. Secondly, LLMs may generate hallucinations or fabricated facts, leading to misinformation. This not only undermines trust but also raises significant concerns about patient safety, posing a major barrier to their practical implementation in healthcare settings.^5^ Third, randomized clinical trials (RCTs) on medical LLMs remained limited, necessitating rigorous validation to assess their practicality. As pointed out by David Ouyang^6^, “We need more RCTs or AI”.

**FIGURE 1:**
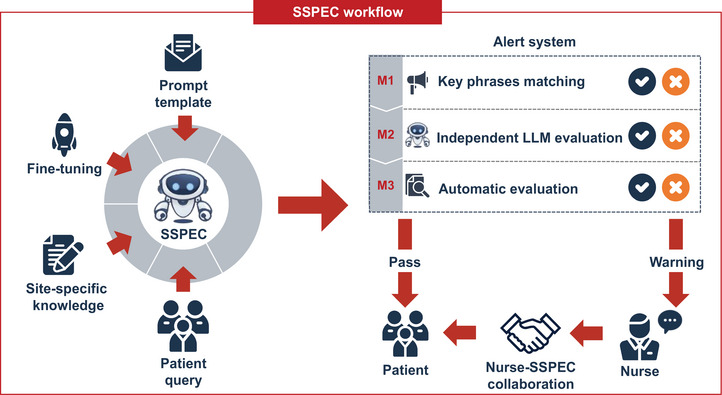
Site‐specific prompt engineering chatbot (SSPEC) framework. The SSPEC framework processes patient queries using a fine‐tuned GPT model (GPT‐3.5 Turbo) enhanced by a prompt template and site‐specific knowledge. SSPEC‐generated responses are evaluated by a three‐tier alert system (M1–M3) before being finalized. If an alert is triggered, the response undergoes nurse review for validation and delivery.

**FIGURE 2:**
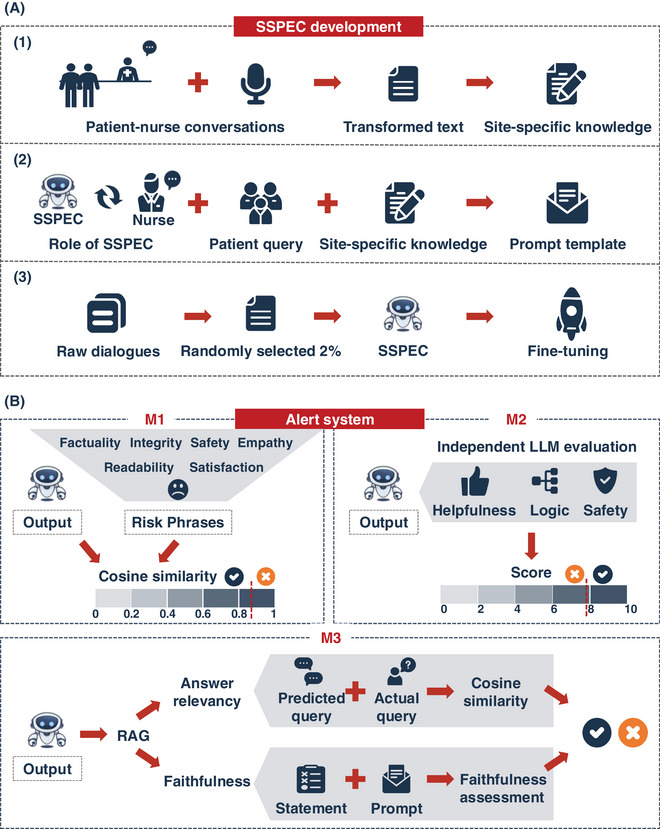
Site‐specific prompt engineering chatbot (SSPEC) development and alert system. (A) The development process of SSPEC: (1) Patient‐nurse conversations are recorded and converted into text to curate site‐specific knowledge; (2) A structured prompt template, consisting of three key components, is integrated into the model. (3) Fine‐tuning is performed by randomly incorporating 2% of raw patient‐nurse dialogues into the training data, enhancing the model's contextual understanding and adaptability. (B) Alert system consists of three modules: M1, cosine similarity is calculated between the model's output and predefined risk phrases to identify potential issues; M2, an independent language model (GPT‐4.0) evaluates the response on three criteria—helpfulness, logic, and safety; and M3, a RAG model assesses faithfulness and answer relevancy to ensure consistency and alignment with the input query.

To address these needs, we developed a site‐specific prompt engineering chatbot (SSPEC) within the “Panoramic Data Collection‐Knowledge Refinement‐Algorithm Enhancement” framework^7^ (Figure 1). This process began with panoramic data collection across each reception site. Next, we incorporated site‐specific knowledge and fine‐tuned the foundation model, GPT‐3.5 Turbo, using a prompt template with three components: Role of SSPEC, Patient Query, and Site‐Specific Knowledge. This approach enriches the foundation model with site‐specific knowledge and enhances its logical reasoning, effectively adapting it to the heterogeneity of medical settings (Figure 2A). Furthermore, the model undergoes iterative refinement through training and clinical trials, enabling SSPEC to achieve superior adaptability and performance compared to the fundamental model in RCT.

To further enhance SSPEC's safety, we developed a knowledge‐aligned alert system to mitigate hallucinations. This system integrates three modules: a key phrases matching module, an independent LLM evaluation module, and a Retrieval‐Augmented Generation (RAG)‐based automatic evaluation module. Key phrases represent hallucination‐related terms (uncertain responses) detected in our real‐world corpora. Semantic similarity between SSPEC responses and key phrases is determined by cosine similarity. The independent LLM evaluation module involves the evaluation of the potential risks in SSPEC responses using an independent model, GPT‐4.0. The RAG‐based automatic evaluation module leverages site‐specific knowledge to construct a knowledge base. It assesses hallucination by measuring the recall rate of responses derived from this knowledge base. If a response fails these evaluations, the system issues an alert, prompting a nurse to review the response, with feedback provided to improve SSPEC's performance (Figure 2B). This nurse‐SSPEC collaboration model effectively mitigated the potential harm of uncertain responses in RCT, with a specificity of 99.8% and a sensitivity of 85.0%.

In populous developing countries with limited healthcare resources, such as China, healthcare providers often experience intense workloads and struggle to maintain high‐quality communication, resulting in inefficiencies and heightened patient‐provider conflicts. A 2016–2019 survey of nearly 40,000 Chinese healthcare providers found that 62% of doctors and 43.8% of nurses were consistently overburdened, a situation further exacerbated by the coronavirus disease 2019 pandemic.^8^ Another survey from 2017 to 2019 across 136 tertiary hospitals showed that the proportion of patient‐nurse conflicts increased from 20.47% to 28.61%.^9^ Despite numerous efforts from the Chinese government, such as the “Healthy China 2030” initiative,^10^ a shortage of qualified healthcare professionals has slowed the progress of these policies. By leveraging generative AI technology to optimize resource allocation and enhance communication, SSPEC has significantly improved healthcare worker efficiency and reduced workload. It has also fostered greater empathy towards patients, effectively lowering patient‐provider conflicts and offering a promising solution to these challenges.

Despite SSPEC's potential, several challenges remain. First, AI must not become a cold barrier between healthcare providers and patients, and over‐reliance by nurses could weaken the essential human connection in patient‐provider interactions. Second, AI acceptance across age groups should be carefully considered to avoid forcing its use, which could overlook the concerns of certain individuals and vulnerable groups. Third, fairness is key: this model should aim to reduce, not exacerbate, healthcare resource disparities between developed and underdeveloped regions.
